# Interrupted Time-Series Analysis to Evaluate the Impact of a Behavioral Change Outpatient Antibiotic Stewardship Intervention

**DOI:** 10.1017/ash.2021.28

**Published:** 2021-07-29

**Authors:** Brittany Morgan, Larissa May, Haylee Bettencourt

## Abstract

**Background:** The Centers for Disease Control and Prevention (CDC) estimates that outpatient settings account for 85%–90% of antibiotic prescriptions in the United States, and ~30% of those prescriptions are unnecessary. One of the most common examples of inappropriate prescribing is for viral upper respiratory infections (URIs). Up to 50% of prescriptions written for URIs are deemed inappropriate, making it an important focus for Antibiotic Stewardship programs. In this study, we evaluated the effect of a behaviorally enhanced quality improvement intervention in reducing inappropriate antibiotic prescribing for viral URIs. **Methods:** A quasi-experimental study assessed the effects of an Antibiotic Stewardship intervention on antibiotic prescribing for viral URIs. The outcome of interest was a change in the number of antibiotics prescribed at each participating clinic over a 1-year preimplementation period and a 2-year postimplementation period. Time trends were analyzed using segmented regression analysis, and a stepped wedge design was used to account for intervention roll-out across clinics. **Results:** From 2017 to 2020, there were 63,028 patient visits in 21 clinic locations. Antibiotics were prescribed an average of 11.5% and 5.8% of visits during the pre- and postimplementation periods, respectively. The most frequently prescribed antibiotic over the study period was azithromycin (n = 3,551), followed by amoxicillin (n = 924). Overall, the intervention was associated with a 46% reduction in antibiotic prescriptions or 0.54 times (*P* = .001) as many inappropriate antibiotics prescribed as before the intervention. There was no significant change in the month-to-month trend in inappropriate prescriptions after the intervention was implemented (*P* = .87). **Conclusions:** Our study demonstrates that a behaviorally enhanced quality improvement intervention to reduce inappropriate prescribing for URI in ambulatory care encounters was successful in reducing potentially inappropriate prescriptions for presumed viral respiratory infections.

**Funding:** No

**Disclosures:** None

